# Associations of Nutrition-Related, Physical, and Social Factors and Their Combinations with Sarcopenia in Community-Dwelling Older Adults: Kashiwa Cohort Study

**DOI:** 10.3390/nu14173544

**Published:** 2022-08-27

**Authors:** Weida Lyu, Tomoki Tanaka, Bo-Kyung Son, Yasuyo Yoshizawa, Masahiro Akishita, Katsuya Iijima

**Affiliations:** 1 Institute of Gerontology, The University of Tokyo, Hongo 7-3-1, Bunkyo-ku, Tokyo 113-0033, Japan; 2 Department of Geriatric Medicine, Graduate School of Medicine, The University of Tokyo, Hongo 7-3-1, Bunkyo-ku, Tokyo 113-0033, Japan; 3 Institute for Future Initiatives, The University of Tokyo, Hongo 7-3-1, Bunkyo-ku, Tokyo 113-0033, Japan; 4 Department of Healthy Life Expectancy, Graduate School of Medicine Juntendo University, Hongo 2-1-1, Bunkyo-ku, Tokyo 113-0033, Japan

**Keywords:** sarcopenia, the versatility of frailty, multi-faceted factors, individual and combined associations

## Abstract

Background: Sarcopenia is a major cause of frailty, which relates to nutrition-related, physical, and social factors. In this study, we aimed to discuss the cross-sectional association of sarcopenia with the above three factors both individually and comprehensively. Methods: Overall, 1257 older adults (≥65 years old) participated in this study. Sarcopenia was determined via the Asian Working Group for Sarcopenia 2019 criteria. The independent variables for nutrition-related, physical, and social factors and especially their criteria for health condition were defined separately. Binomial logistic regression analysis was carried out to testify the associations of sarcopenia with three factors individually and in combination. Results: The mean age was 74.6 (±5.5), and women were 47.7%. Sarcopenia prevalence was 7.5%. Participants who did not meet the criteria of nutritional health, physical fitness, or social robustness independently had significant associations with a higher adjusted odds ratio (aOR) of sarcopenia or its indices of lower grip strength, muscle mass, or gait speed. In comparison to participants meeting three criteria, those who met two, one, or none showed (marginally) significant association with increased aOR for sarcopenia (aOR (95% confidence interval)): two: 1.97 (0.84–4.64); one: 2.35 (1.00–5.23); none: 5.52 (2.30–13.23). Conclusions: Comprehensive countermeasures with the above three factors are indispensable for sarcopenia prevention.

## 1. Introduction

Sarcopenia is an age-linked skeletal muscle related issue involving the skeletal muscle mass and strength loss as well as a physical function decrease [1,2]. According to previous studies, muscle mass shrinks by 1–2% per year from the 50th year of life, and muscle strength decreases by 1.5–3% annually between the ages of 50 and 60 and 3% thereafter [3]. Sarcopenia has been closely connected with outcomes such as falls, disability, hospitalization, and even mortality especially among older adults [4,5]. Moreover, from the healthcare viewpoint, sarcopenia results in an increased financial burden [6,7]. These findings indicate that sarcopenia is accompanied by severe clinical aftereffect and is regarded as a public health threat [6]. With the rapid and continuous increase in the aging population and sarcopenia prevalence worldwide, especially in Japan, sarcopenia prevention has been a healthcare priority in geriatrics.

In recent years, countermeasures against sarcopenia have attracted increasing attention, especially from the viewpoint of daily life habits, including nutrition [8,9], oral health [10], physical activity [11,12], and sociality [13]. Moreover, the combination effect, especially the combination of exercise and nutrition (amino acids, tea catechins, or vitamin D), has been proven to be a benefit to physical function and even sarcopenia among older people [14,15,16]. Moreover, better dietary quality combined with active physical activity and a healthy lifestyle is connected with a low sarcopenic obesity prevalence [17].

However, discussions on the combined effects of social factors with nutrition-related or physical factors or both are quite few. In our recent study, we discussed the association of frailty with the above three factors, which indicated that people who met the above three factors’ criteria showed the lowest frailty prevalence and in increasing tendency with a decreasing quantity of met criteria [18]. Although there are still some common aspects between frailty and sarcopenia, in which they are both defined by the physical function decline of gait speed and muscle strength with aging [19], overlaps do exist in which frailty is a multiple-physiologic deterioration accompanied by worse vulnerability to stressors, while sarcopenia is more focused on muscles [20]. Therefore, in this study, we further investigated the cross-sectional associations of sarcopenia with nutrition-related, physical activity, and social factors comprehensively as well as individually among older adults. In this study, we hypothesized that the older adults who met the criteria of nutritional health, physical fitness, or social robustness individually showed lower sarcopenia and sarcopenia indices (lower muscle mass, muscle strength, or gait speed) prevalence. Moreover, with the decreasing of the quantity of the met criteria (from 3 to 0), sarcopenia prevalence also showed increase trend.

## 2. Materials and Methods

### 2.1. Study Design and Participants

The Kashiwa cohort study randomly selected 12,000 community-dwelling older adults (≥65-years-old) in Kashiwa City, Japan, at the baseline time of 2012; consequently, 2044 older adults (1013 men and 1031 women) participated in the survey. This study included 1308 participants attending the follow-up survey of Kashiwa cohort study from September to November 2016. The exclusion criteria contain the following: (a) cognitive dysfunction (mini-mental state examination (MMSE), ≤18); (b) participants who are using pacemakers because the muscle mass will be assessed by bioelectrical impedance analysis, and it has potential interference to pacemaker; and (c) participants with missing data. The overall flow of this study is shown as Figure S1. This study was approved by the ethics committee (#18-255) of the University of Tokyo and conducted under the guidelines of the Declaration of Helsinki [21]. Participants’ data were anonymized with ID numbers only for further analysis.

### 2.2. Measurements

#### 2.2.1. Outcome

The outcome was sarcopenia, defined based on the Asian Working Group for Sarcopenia 2019 (AWGS2019) and consisting of three components: muscle mass, strength, and physical function. By the criteria of AWGS2019, people with low muscle mass as well as weak muscle strength or poor physical function are considered to have sarcopenia, while the others are categorized as “non-sarcopenia” [22]. The measurement methods and criteria for each component are as follows:

The appendicular skeletal muscle mass index (ASMI) for the muscle mass was assessed by bioelectrical impedance analysis using the InBody 430 (InBody Japan, Tokyo, Japan) by adjusting for the square of the height. Low muscle mass was described with ASMI as <7.0 kg/m^2^ for men and <5.7 kg/m^2^ for women [22].

Muscle strength (dominant hand’s grip strength in this study) was evaluated with a grip dynamometer (Grip D; Takei Scientific Instruments, Niigata, Japan) [23]. The better values of two tests assessed by the support of trained testers were used. The cut-off of grip strength was: men, <28 kg; women, <18 kg [22].

Physical function (usual gait speed in this study) was determined by trained testers. On an 11 m straight line, participants’ walking time between 3 m and 8 m from the start line was used for the gait speed calculation [23]. Gait speed less than 1.0 m/s was defined as low physical function [22].

#### 2.2.2. Independent Variables

The following three factors have been described in our previous study [18] with the following concerns: (1) Every item was connected with the daily living behaviors; and (2) the proportion of each factor was maintained at approximately 40–60% by modifying the combinations of each item via “or/and” to make each criterion be moderate to practice in difficulty in community-dwelling older adults.

(1) Nutrition-related factors:

Nutrition-related factors include the balance diet and oral functions. Balanced diet contains the food diversity, daily protein, and vegetable intake. Food diversity score are calculated based on 10 types of food. Total scores are from 0 to 10 [24], and cut-off was set up as ≥4 [25]. Moreover, protein and vegetable intake conditions were investigated because of their importance in sarcopenia prevention [26,27]. Oral health has been indicated to be associated with sarcopenia [28], and oral health has been shown to be connected with the nutrition aspect; therefore, we considered oral functions as one item contained in nutrition-related factors [29].

Based on the above, nutrition-related factors are described as following: (1) food diversity score ≥ 4, (2) almost daily consumption of meat or fish and vegetables, and (3) whether they eat hard foods such as squid jerky or pickled radish. The criteria for “nutritional health” are defined as meeting (1) or (2) and (3).

(2) Physical factors:

Physical factors were defined via the physical habits and awareness with 3 items from the “Standard Health Checkup and Counseling Guidance Program” [30]: (1) exercise for 30 min or more per day, twice a week for more than a year; (2) walking/equivalent physical factors for 1 h or more per day; and (3) walking speed faster than most people of the same sex and age. The criteria for “physical fitness” were defined as any two or three items met.

(3) Social factors:

Social factors consist of three items: social organizational participation, social support, and social networks. Social organizational participation was described by the quantity of the 7 organizations that participants joined, and the cutoff was ≥1 [18]. Social support was defined using the 2-Way Social Support Scale, of which the total scores are from 0 to 4, and the cut-off was 4 [31]. Social networks were defined using the Lubben Social Network Scale (LSNS-6) with 6 questions [32]. The total scores are from 0 to 30, and cut-off was ≤12, which is regarded as being socially isolated according to previous study [33].

The criteria for “social robustness” was whether participants met all three items.

(4) Three factors’ combination:

First, according to the quantity of the met criteria described above, 4 groups were divided into: satisfied quantity 3 (met all three factors’ criteria), satisfied quantity 2 (met any two), satisfied quantity 1 (met any one), and satisfied quantity 0 (met none).

Furthermore, 2 groups were differentiated based on whether all three factors’ criteria were met or not: satisfied integrality, yes (met all); satisfied integrality, no (met less than three).

#### 2.2.3. Covariates

The covariates contained participants’ age, sex, body mass index (BMI), ASMI, living with others/alone, depressive symptoms measured by the Geriatric Depression Scale-15 (GDS-15) [34], and chronic conditions (hypertension, chronic renal failure, diabetes mellitus, heart disease, osteoporosis, hyperlipidemia, stroke, and malignant neoplasm) checked by a standardized questionnaire.

### 2.3. Statistical Analysis

First, background characteristics comparisons were conducted between non-sarcopenia and sarcopenia participants using an unpaired *t*-test/Mann–Whitney U test for continuous variables and a chi-square (χ2)/Fisher’s exact test for categorical variables. The percentages of each factor and their items contained among all the participants as well as their corresponding percentages via sarcopenia status were evaluated and then tested by the chi-square (χ2) test.

Individual associations of three factors with sarcopenia or sarcopenia indices (grip strength, muscle mass, and gait speed) were discussed by binomial logistic regression analysis, with the participants meeting the criteria as the reference.

Furthermore, binomial logistic regression analysis was further run to explore the comprehensive association of satisfied quantity (the quantity of met criteria, 4 groups) or satisfied integrality (if all three 3 criteria were met or not, 2 groups) with sarcopenia, with the participants of satisfied quantity 3/satisfied integrality, yes as the reference. The adjusted odds ratio (aOR) and 95% confidence intervals (CIs) were calculated.

Furthermore, to testify if the sample size was appropriate for the above binomial logistic regression analysis by avoiding type 1 error (the error of rejecting a null hypothesis (H0) when it is true) and type 2 error (the error of accepting a null hypothesis when the alternative hypothesis (H1) is true), statistical power calculation for logistic regression was conducted with the covariates described above. All analyses were conducted by IBM SPSS Statistics version 28 (IBM Japan, Tokyo, Japan), and *p-Values* < 0.05 were considered statistically significant.

## 3. Results

Among 1308 participants attending the follow-up survey in 2016, 51 were excluded based on the criteria described above (Figure S1). The mean age of the 1257 participants was 74.6 ± 5.5, and the women were 47.7%. Ninety-four patients (7.5%) with sarcopenia were identified. The comparison of participants’ background characteristics was carried out between the non-sarcopenia and sarcopenia groups. Participants with sarcopenia were older, with lower BMI and ASMI, usual gait speed and grip strength, and a higher osteoporosis and heart disease ratio (Table 1).

The relative proportions of nutrition-related, physical, and social factors and their corresponding items overall and between the non-sarcopenia and sarcopenia groups are shown in Table 2. Compared to participants with sarcopenia, those non-sarcopenia participants showed a significantly higher proportion of nutrition-related, physical, and social factors met. Specifically, non-sarcopenia participants had a higher proportion of healthy oral functions, good physical habits, awareness of physical function, and social support condition.

The associations between sarcopenia and individual nutrition-related, physical, and social factors were individually investigated (Table 3). Nutrition-related and physical factors were significantly associated with greater crude and adjusted OR (aOR) of sarcopenia in participants who did not meet the criteria of nutritional health or physical fitness. For social factors, meeting no criteria of social robustness had a significant association with greater crude OR with sarcopenia, while the significant difference disappeared in aOR adjusted for all covariates. The results indicated that nutrition-related and physical factors were directly and significantly associated with sarcopenia.

In addition to sarcopenia, the associations between sarcopenia indices and nutrition-related, physical, and social factors were discussed separately. The results indicated that meeting no criteria of nutritional health was significantly associated with a greater aOR of lower grip strength. For the physical factors, participants who did not meet the criteria of physical fitness were significantly connected with greater aOR on lower grip strength, muscle mass, and gait speed. Regarding social factors, we found that meeting no criteria of social robustness was associated with a greater aOR for lower gait speed (Table 4). Combined with the results shown in Table 3; Table 4, we found that nutrition-related factors were directly associated with sarcopenia because of their close association with muscle strength. Physical factors showed a direct and significant association with sarcopenia due to their wide association with muscle mass/strength and physical function. Furthermore, although social factors showed no significant association with sarcopenia, their strong association with gait speed indicated that they were, to some degree, indirectly associated with sarcopenia.

The comprehensive relationship between these three factors and sarcopenia was investigated, and the results are shown in Table 5. The association between satisfied quantities (quantity of met criteria) and sarcopenia was discussed. Compared to who met all three criteria (satisfied quantity 3), the groups of who met any two, any one, or none (satisfied quantity 2, 1, 0) showed significant association with increased aOR for sarcopenia (Table 5). Moreover, in comparison to the participants of satisfied integrality, yes, those of satisfied integrality, no also showed significantly greater aOR for sarcopenia. We further conducted the effect size and power calculation analysis. For the given parameters, under the given sample size of 1257 observations and computed using alpha of 0.050 (probability of type 1 error was 5%), R^2^ was 0.120 (adjusted R^2^ was 0.109), and observed power level was 0.975 (probability of type 2 error was 2.5%) (*p* < 0.001), which indicated that the sample size in this study was appropriated for the above binomial logistic regression analysis.

## 4. Discussion

In this study, we investigated the associations of nutrition-related, physical, and social factors with sarcopenia individually and, further, their combinations in older adults. We found that older adults who did not meet the criteria of nutritional health, physical fitness, or social robustness showed significantly greater aOR for sarcopenia or its indices. Furthermore, in this study, we would like to emphasize the association of three factors’ combination with sarcopenia, especially since, according to the results, compared to the participants who meet three factors’ criteria, even two criteria also showed marginally significantly OR aOR. Moreover, with the quantity of met criteria decreasing from three to none (satisfied quantity 3 to 0), OR or aOR for sarcopenia also showed an increasing trend. We also confirmed the significant association of the combination of the three factors with sarcopenia by two-group comparisons (satisfied integrality, yes vs. no). Participants of satisfied integrality, no showed significantly greater crude and aOR.

We defined the nutrition-related factors based on food diversity, protein and vegetable intake, and oral functions. This is because that balanced dietary variety, appropriate dairy protein intake, and vegetable and fruit consumption have been reported to be benefit to grip strength [25,35] and sarcopenia prevention in older adults. Moreover, the combination of protein with vitamins could boost muscle strength and anabolic markers in sarcopenia older adults according to a double-blind, randomized controlled trial [36]. Regarding oral health, Tanaka [10] reported that oral frailty might potentially predict new-onset sarcopenia. Poor oral health may represent a risk factor for sarcopenia by restricting both food selection and nutrient intake in older adults [37]. In this study, nutrition-related factors showed closely associated with sarcopenia and one of its indices of grip strength; however, no significant association was observed between nutrition-related factors and muscle mass or gait speed. The above results do not rule out the possible positive effects of nutrition-related factors on muscle or physical function because there are still previous studies that indicated that it is not nutrition or exercise alone but the combination of exercise and protein intake that showed significant improvement in muscle quality and strength [38,39]. Moreover, for the participants who met the criteria of nutrition-related factors, we cannot make clear conclusions about the met condition of physical or social factors. On the other words, the lack of the other two factors might be one of the reasons leading to the above results. This result also, to some degree, reflected the importance of multi-factors’ combination.

The physical factors in this study were proven to be directly associated with sarcopenia and three sarcopenia indices. Exercise as a remedy is beneficial in preventing sarcopenia and decreasing physical function [11,12]. For example, it has been advocated for all ages to carry out appropriate physical activity at least half an hour per day [40].

The social factors play the key role in sarcopenia. It has been suggested that social engagement improves sarcopenia indices and then reduces sarcopenia development risk by influencing physical, oral, psychological, and nutritional status [13]. Moreover, social participation and social networks have also been proven to be associated with physical functions of muscle strength as well as gait speed [41,42,43].

From the discussions above, we could find that, for community-dwelling older adults, it is doubtlessly essential to keep good habits and behavior in each of the above factors. As above, we further investigated the association of sarcopenia with three factors’ combination. According to previous study, especially the combination of exercise and nutrition have been addressed. For example, amino acids, tea catechins, and vitamin D assembled with exercises such as the balance or gait function training have also been suggested to improve older adults’ muscle mass/strength and gait speed in older adults [14,15,16]. In recent years, discussions about the association of sarcopenia with social factors have begun to come into focus according to previous studies; however, the combined effect of social factors with nutrition-related or physical factors or both is still deficient. According to our previous study, multidimensional social engagement affects factors such as physical, oral, psychological, and nutritional status, potentially decreasing new-onset sarcopenia risks [10]. Additionally, in our recent study, we discussed the association of frailty with three factors, which indicated that meeting all three criteria showed the lowest frailty prevalence among older adults [18]. Considering the commonalities between frailty and sarcopenia, which are both characterized by the physical function impairment of gait speed and muscle strength with aging [19], as well as the relative discussions on the association of each three factors with sarcopenia individually, we have sufficient reason to believe that sarcopenia is closely connected with the three factors and especially their combination.

The findings indicated that it is essential to keep healthy life behaviors and habits of the above three factors as much as possible for community-dwelling older adults from the viewpoint of sarcopenia prevention. In this study, the older adults who met all of three criteria comprised only 19.1%, which means that the majority of older adults still have space for improvement. However, we have to say that it is, to some degree, difficult to change the lifestyle and daily habits of older adults that have been in practice for years. From this point, based on the findings in this study, we will focus on the exploration of developing comprehensive, practicable, and continuable sarcopenia countermeasures in our future work. To achieve this aim, we would like to go deep among communities and older adults to explore their real needs and gain their valuable experience on sarcopenia prevention.

There were several limitations in this study. First, the discussion on sex differences was not conducted due to a relatively small sample size. Second, the causal associations between sarcopenia and the three factors and their combinations are still unclear because of the cross-sectional design of the study. Third, uncontrolled or unmeasured confounders may exist because of the observational nature of this study. Fourth, some of the investigations about nutrition-related and social factors are subjective in nature and may introduce recall bias, especially considering the older population in this study. Based on the results of this study, although longitudinal studies are needed to test the long-term impact of these three factors on sarcopenia, comprehensive practice might benefit sarcopenia prevention.

## 5. Conclusions

Sarcopenia is greatly involved when older adults fall into a frail or nursing care state. Based on our previous study, which suggested the relationship of frailty with the nutrition-related, physical, and social factors was proven, we further proved the association of sarcopenia, a major cause of frailty, with these three factors both individually and in combination. The participants meeting all three criteria had the lowest sarcopenia prevalence, which also showed an increasing trend with a decrease in the met criteria. Based on the findings in this study, we suggest that rather than a single approach, comprehensive countermeasures including the above three factors are indeed essential for sarcopenia prevention in community-dwelling older adults.

## Figures and Tables

**Table 1:** Participants’ background according to sarcopenia Status.

	Overall		Non-Sarcopenia		Sarcopenia		*p*-Value ^§^
*No. of participants*	1257		1163	(92.5%)	94	(7.5%)	*-*
*Basic attributes*							
Age, years	74.6	(±5.5)	74.3	(±5.3)	79.3	(±6.0)	*0.001 ^a^*
Sex, women	599	(47.7%)	556	(47.8%)	43	(45.7%)	*0.700 ^b^*
Education level, years	12.9	(±2.8)	12.9	(±2.8)	12.6	(±2.9)	*0.340 ^a^*
Living arrangements, living alone	166	(13.2%)	149	(12.8%)	17	(18.1%)	*0.146 ^b^*
*Physical and psychological attributes*							
BMI, kg/m^2^	22.3	(±2.9)	22.5	(±2.9)	20.1	(±2.5)	*<0.001 ^a^*
Men	22.7	(±2.7)	22.9	(±2.7)	20.4	(±2.2)	*<0.001 ^a^*
Women	21.9	(±3.1)	22.0	(±3.0)	19.8	(±2.9)	*<0.001 ^a^*
ASMI, kg/m^2^	6.7	(±1.0)	6.7	(±1.0)	5.8	(±0.8)	*<0.001 ^a^*
Men	7.3	(±0.7)	7.4	(±0.6)	6.3	(±0.6)	*<0.001 ^a^*
Women	5.9	(±0.7)	6.0	(±0.6)	5.1	(±0.4)	*<0.001 ^a^*
Grip strength, kg	29.7	(±8.2)	30.3	(±8.0)	21.2	(±5.0)	*<0.001 ^a^*
Men	35.5	(±6.3)	36.4	(±5.7)	25.1	(±2.8)	*<0.001 ^a^*
Women	23.2	(±4.3)	23.7	(±4.0)	16.7	(±2.8)	*<0.001 ^a^*
Usual gait speed, m/s	1.4	(±0.2)	1.5	(±0.2)	1.3	(±0.3)	*<0.001 ^a^*
Men	1.5	(±0.2)	1.5	(±0.2)	1.2	(±0.3)	*<0.001 ^a^*
Women	1.4	(±0.2)	1.4	(±0.2)	1.3	(±0.3)	*<0.001 ^a^*
MMSE score	29.0	(28.0–30.0)	29.0	(28.0–30.0)	28.0	(27.0–29.3)	*<0.001 ^c^*
GDS-15 score	2.0	(1.0–5.0)	2.0	(1.0–5.0)	4.0	(2.0–6.3)	*<0.001 ^c^*
*Present chronic conditions*							
Hypertension	579	(46.1%)	533	(45.8%)	46	(48.9%)	*0.561 ^b^*
Diabetes mellitus	153	(12.2%)	144	(12.4%)	9	(9.6%)	*0.423 ^b^*
Osteoporosis	137	(10.9%)	118	(10.1%)	19	(20.2%)	*0.003 ^b^*
Hyperlipidemia	448	(35.6%)	422	(36.3%)	26	(27.7%)	*0.093 ^b^*
Chronic renal failure	8	(0.6%)	7	(0.6%)	1	(1.1%)	*0.588 ^b^*
Stroke	84	(6.7%)	75	(6.4%)	9	(9.6%)	*0.243 ^b^*
Heart disease	218	(17.3%)	192	(16.5%)	26	(27.7%)	*0.006 ^d^*
Malignant neoplasm	200	(15.9%)	186	(16.0%)	14	(14.9%)	*0.779 ^b^*

Notes: BMI, body mass index; ASMI, Appendicular Skeletal Muscle Mass Index; MMSE, Mini-Mental State Examination; GDS-15, Geriatric Depression Scale-15. ^§^. *p*-values were calculated as followings: ^a^ Unpaired *t*-test; ^b^ Pearson’s chi-square test; ^c^ Mann–Whitney U test.; ^d^ Fisher’s exact test.

**Table 2:** Proportion of each and total items of nutrition-related, physical, and social factors as well as their corresponding proportion according to sarcopenia status (n = 1257).

Items	Overall n (%)	Non-Sarcopenia n (%)	Sarcopenia n (%)	*p*-Value ^§^
Nutrition-related factors				
(1) Food diversity score ≥ 4	750 (59.7%)	700 (60.2%)	50 (53.2%)	*0.183*
(2) Almost daily consumption of meat or fish and vegetables, yes	465 (37.0%)	434 (37.3%)	31 (33.0%)	*0.402*
(3) Can you eat hard foods like squid jerky or pickled radish, yes	1021 (81.2%)	958 (82.4%)	63 (67.0%)	*<0.001*
Criteria of nutrition-related factors: (1) or (2) and (3)	643 (51.2%)	607 (52.2%)	36 (38.3%)	*0.010*
Physical factors				
(1) Exercise for 30 min or more per day, twice a week for more than one year, yes	720 (57.3%)	685 (58.9%)	35 (37.2%)	*<0.001*
(2) Walking (or equivalent physical factors) for 1 h or more per day, yes	834 (66.3%)	779 (67.0%)	55 (58.5%)	*0.095*
(3) Gait speed is faster than most people with the same sex and age, yes	783 (62.3%)	740 (63.6%)	43 (45.7%)	*<0.001*
Criteria of physical factors: any 2 or 3 items	797 (63.4%)	757 (65.1%)	40 (42.6%)	*<0.001*
Social factors				
(1) Participate in one or more activities of organizations, yes	1098 (87.4%)	1016 (92.5%)	82 (87.2%)	*0.972*
(2) Social support score (provision and receipt), score = 4	692 (55.1%)	654 (56.2%)	38 (40.4%)	*0.003*
(3) Social network score, LSNS-6 score ≥ 12	1147 (91.2%)	1060 (91.1%)	87 (92.6%)	*0.642*
Criteria of social factors: (1) and (2) and (3)	582 (46.3%)	549 (47.2%)	33 (35.1%)	*0.024*

^§^, *p*-values were calculated by chi-square test.

**Table 3:** The association of each factor with sarcopenia by binomial logistic regression analysis (n = 1257).

	Sarcopenia n (%)	Crude OR (95% CI)	*p*-Value	aOR ^a^ (95% CI)	*p*-Value
Nutrition-related factors					
Criteria met	36 (5.6%)	1.00 (Reference)		1.00 (Reference)	
Criteria not met	58 (9.4%)	1.76 (1.14–2.71)	*0.010*	2.03 (1.26–3.29)	*0.004*
Physical factors					
Criteria met	40 (5.0%)	1.00 (Reference)		1.00 (Reference)	
Criteria not met	54 (11.7%)	2.52 (1.64–3.86)	*<0.001*	2.39 (1.48–3.86)	*<0.001*
Social factors					
Criteria met	33 (5.7%)	1.00 (Reference)		1.00 (Reference)	
Criteria not met	61 (9.0%)	1.65 (1.07–2.56)	*0.025*	1.25 (0.76–2.05)	*0.371*

Notes: OR, odds ratio with no covariates; 95%CI, 95% confidence interval; aOR, adjusted odds ratio; 95%CI, 95% confidence interval. ^a^ aOR, odds ratio adjusted by age, sex, living arrangements (living alone/living with others), education level (years), BMI, chronic conditions (hypertension, diabetes mellitus, osteoporosis, hyperlipidemia, chronic renal failure, stroke, heart disease, and malignant neoplasm), and GDS-15.

**Table 4:** The association of each factor with sarcopenia indices by binomial logistic regression analysis (n = 1257).

	Lower Grip Strength			Lower Muscle Mass			Lower Gait Speed		
	Proportion n (%)	aOR ^a^ (95% CI)	*p*-Value	Proportion n (%)	aOR ^a^ (95% CI)	*p*-Value	Proportion n (%)	aOR ^a^ (95% CI)	*p*-Value
Nutrition-related factors									
Criteria met	51 (7.9%)	1.00 (Reference)		204 (35.0%)	1.00 (Reference)		21 (2.5%)	1.00 (Reference)	
Criteria not met	72 (11.7%)	1.78 (1.18–2.69)	*0.006*	215 (31.7%)	1.23 (0.91–1.67)	*0.184*	15 (3.3%)	1.14 (0.55–2.37)	*0.735*
Physical factors									
Criteria met	62 (7.8%)	1.00 (Reference)		228 (28.6%)	1.00 (Reference)		12 (1.5%)	1.00 (Reference)	
Criteria not met	61 (13.3%)	1.77 (1.16–2.69)	*0.008*	191 (41.5%)	2.35 (1.69–3.27)	*<0.001*	24 (5.2%)	2.44 (1.13–5.26)	*0.023*
Social factors									
Criteria met	45 (7.7%)	1.00 (Reference)		173 (29.7%)	1.00 (Reference)		7 (1.2%)	1.00 (Reference)	
Criteria not met	78 (11.6%)	1.25 (0.82–1.92)	*0.299*	246 (36.4%)	1.11 (0.81–1.51)	*0.521*	29 (4.3%)	2.64 (1.08–6.44)	*0.033*

Notes: OR, odds ratio with no covariates; 95%CI, 95% confidence interval; aOR, adjusted odds ratio; 95%CI, 95% confidence interval. ^a^ aOR, odds ratio adjusted by age, sex, living arrangements (living alone/living with others), education level (years), BMI, chronic conditions (hypertension, diabetes mellitus, osteoporosis, hyperlipidemia, chronic renal failure, stroke, heart disease and malignant neoplasm), and GDS-15.

**Table 5:** The associations between the quantity of met criteria with sarcopenia status by binomial logistic regression analysis (n = 1257).

Satisfied Quantity ^a^	Overall	Sarcopenia n (%)	Crude OR (95% CI)	*p*-Value	aOR ^b^ (95% CI)	*p*-Value	Satisfied Integrality ^b^	Crude OR (95% CI)	*p*-Value	aOR ^c^ (95% CI)	*p*-Value
Three	240 (19.1%)	7 (2.9%)	1.00 (Reference)		1.00 (Reference)		Yes	1.00 (Reference)		1.00 (Reference)	
Two	453 (36.3%)	29 (6.4%)	2.26 (0.98–5.24)	*0.057*	2.25 (0.91–5.54)	*0.078*	No	2.67 (1.21–5.93)	*0.004*	2.83 (1.21–6.58)	*0.016*
One	390 (31.0%)	30 (7.7%)	2.77 (1.20–6.42)	*0.017*	2.42 (0.97–6.02)	*0.058*					
Zero	171 (13.6%)	28 (16.4%)	6.52 (2.78–15.31)	*<0.001*	6.35 (2.46–16.40)	*<0.001*					

Notes: OR, odds ratio with no covariates; 95%CI, 95% confidence interval; aOR, adjusted odds ratio; 95%CI, 95% confidence interval. ^a^ the quantity of met criteria of the three factors. ^b^ if all the three factors’ criteria were met or not. ^c^ aOR, odds ratio adjusted by age, sex, living arrangements (living alone/living with others), education level (years), BMI, chronic conditions (hypertension, diabetes mellitus, osteoporosis, hyperlipidemia, chronic renal failure, stroke, heart disease and malignant neoplasm), and GDS-15.

## Data Availability

Not applicable.

## Supplementary Materials

The following supporting information can be downloaded at: https://www.mdpi.com/article/10.3390/nu14173544/s1, Figure S1: Flow diagram.
