# Supplementary figures and images for: Associations of Nutrition-Related, Physical, and Social Factors and Their Combinations with Sarcopenia in Community-Dwelling Older Adults: Kashiwa Cohort Study
Source: Nutrients. 2022 Aug 27;14(17):3544. doi: 10.3390/nu14173544 (PMC9460705; doi:10.3390/nu14173544)

**Supplementary Materials:** Figure S1: Flow diagram of the study.

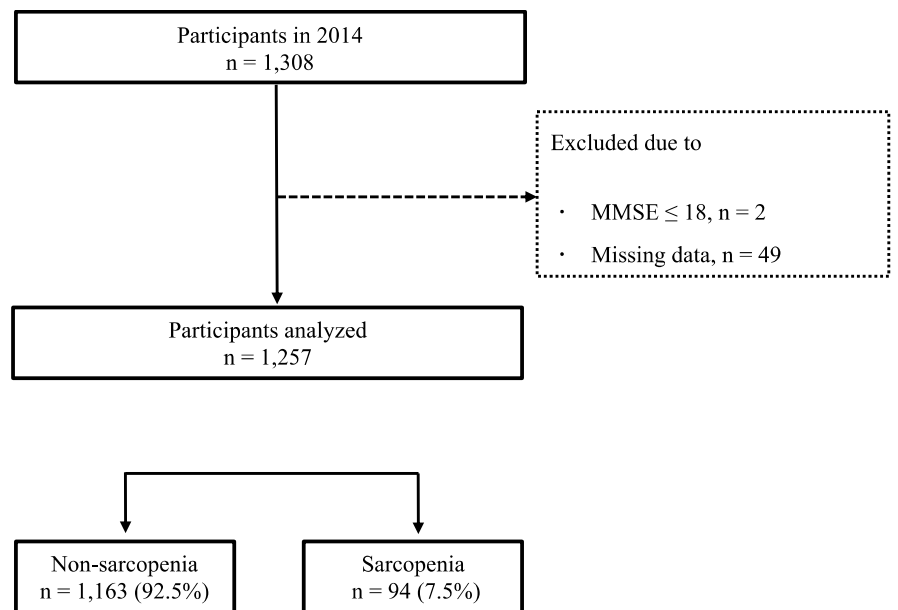

Supplement: Supplementary file 1 [file nutrients-14-03544-s001.zip › nutrients-1857103-supplementary.pdf]
